# Genomic recovery from rare terrestrial microbes enabled by DNA-based GC-fractionation

**DOI:** 10.1093/ismeco/ycaf152

**Published:** 2025-09-03

**Authors:** Paul O Sheridan, Yiyu Meng, Dylan Bodington, David Coutts, Tom A Williams, Cécile Gubry-Rangin

**Affiliations:** School of Biological Sciences, University of Aberdeen, Aberdeen AB24 3UU, United Kingdom; School of Biological and Chemical Sciences, University of Galway, Galway H91 TK33, Ireland; School of Biological Sciences, University of Aberdeen, Aberdeen AB24 3UU, United Kingdom; School of Biological Sciences, University of Aberdeen, Aberdeen AB24 3UU, United Kingdom; School of Biological Sciences, University of Aberdeen, Aberdeen AB24 3UU, United Kingdom; School of Biological Sciences, University of Bristol, Bristol BS81TQ, United Kingdom; School of Biological Sciences, University of Aberdeen, Aberdeen AB24 3UU, United Kingdom

**Keywords:** GC-content, bisbenzimide, genome, MAG, 16S rRNA gene, rare, low abundance, bacteria, archaea, AOA

## Abstract

Genome reconstruction from metagenomic samples has dramatically increased our understanding of uncultivated lineages of life. However, untargeted metagenomic sequencing is biased towards the more abundant microbes, neglecting less abundant lineages playing important ecological roles, such as the ammonia-oxidising archaea. Here, we demonstrate that separating soil molecular DNA using a bisbenzimide-CsCl guanine-cytosine (GC)-content-based DNA fractionation approach separates microbial DNA along a GC-content gradient. The fractions from both extremes of the GC-content gradient possess different 16S rRNA gene composition than the original unfractionated DNA. The high diversity in the lower GC-content fractions (< 45%) contrasts with the higher DNA abundance in the higher GC-content fractions (50%–70%), highlighting the low GC fractions as an enriched source of rare microbe DNA. Metagenomic sequencing of specific low- and high-GC fractions enabled the reconstruction of 204 taxonomically diverse metagenome-assembled genomes from 31 microbial phyla, with at least 63 of these originating from rare (< 0.1% relative abundance) or very rare (< 0.01% relative abundance) microbial families. Therefore, this approach facilitates genomic assembly of rare taxa in resulting pseudo-communities. Ultimately, this technique enables a semi-targeted metagenomic approach to recover genomes from low-abundance microbes with GC-contents that differ significantly from the environmental microbial community of interest. As mounting evidence suggests that rare microbes drive critical ecosystem functions, this approach will facilitate a deeper understanding of their metabolic potential in the environment.

## Graphical Abstract

**Graphical Abstract:**
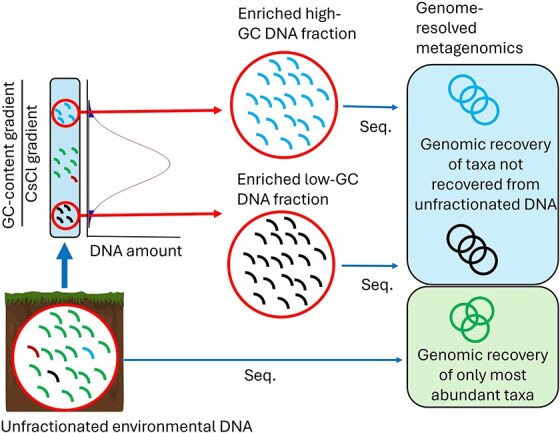

## Introduction

The ability to reconstruct genome sequences from shotgun metagenomic reads has greatly increased the genomic representation of the tree of life [1, 2], enabling metabolic prediction of many uncultivated microbes. However, complex microbial communities comprise hundreds to thousands of taxonomic phylotypes at vastly uneven abundances, with a high number of rare species [3]. In typical metagenomic experiments, most reads will be derived from abundant microorganisms [4–6], with rare microbes being poorly represented in the resulting genomic datasets. This is a problem because low-abundance taxa may perform a critical role in ecosystem function, health, and stability [3]. For example, despite their low abundance, the ammonia-oxidising archaea (AOA) perform the first step of the nitrification pathway and, therefore, are key players in the nitrogen cycle in multiple environments, including soil, marine, freshwater, sediment, marine sponge, and geothermal ecosystems [7–9]. In addition, low-abundance microbes contribute to the temporal community dynamics of an ecosystem [3], as they represent a seedbank of diversity which can be reactivated from dormancy in response to environmental change [10]. Therefore, reconstructing genomes of low-abundance taxa is important to better understand the role and ecosystem function of those organisms, even enabling genomics-resolved cultivation [11].

Reconstructing medium- to high-quality [12] draft genomes (metagenomics-assembled genomes, MAGs) of low-abundance taxa can theoretically be achieved by extremely high sequencing depth and massive computational resources. However, DNA enrichment approaches offer several technical benefits, including enhanced sequencing sensitivity and reduced sequencing and assembly costs, which are aligned with a lower required sequencing depth. Multiple selective sequencing approaches have been previously developed to enrich the DNA of low-abundance taxa. For example, methylation-guided enrichment approaches selectively enrich the microbial DNA by removing the host DNA based on its higher methylation level [13]. In addition, targeted-sequencing approaches enrich the target DNA during the library preparation, including with CRISPR-Cas9 enrichment or hybrid capture [14]. These later approaches require prior knowledge of the targeted community, which is often difficult to obtain in many underexplored ecosystems. Recently, selective nanopore sequencing and associated bioinformatic scripts were used to achieve efficient enrichment of rare taxa in diverse ecosystems [15] paving the way for computational development to acquire genomes of low-abundance taxa. However, the development of empirical approaches to enrich the microbial DNA of low-abundance taxa without prior knowledge of the community’s genomic content would advance the field of rare microbiomes.

**Figure 1:**
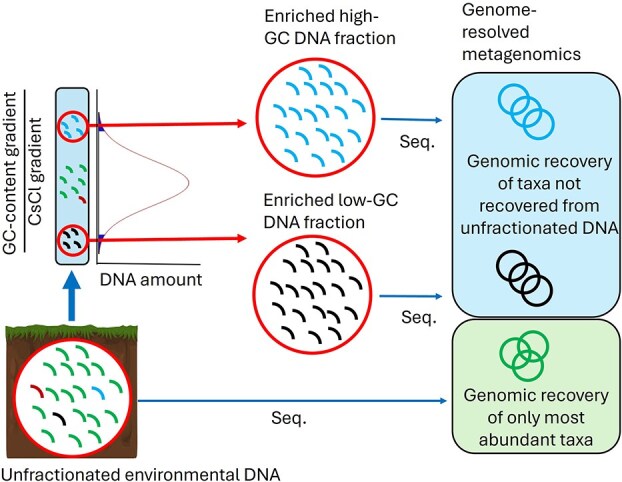
Conceptual representation of the bisbenzimide-CsCl DNA fractionation approach for genomic recovery of non-abundant microbes.

To fill this gap of knowledge, we present a wet-lab approach which enables DNA separation based on the guanine-cytosine (GC) content of the organismal DNA. We hypothesized that GC-content-based DNA fractionation reduces the microbial community complexity into restricted GC-content pseudo-communities before sequencing. Additionally, the non-intercalating DNA dye bisbenzimide (also known as Hoechst 33258) preferentially binds to A + T DNA regions, enabling greater separation of DNA fragments based on GC-content and determined buoyant density [16]. As most soil environmental DNA tends to have high GC-content [17], the proposed approach should enable the enrichment of DNA from rare, low GC-content genomes and the selective sequencing of low GC-content DNA organisms. Bisbenzimide-enhanced GC-content-based DNA fractionation has been demonstrated in diverse ecosystems including plants [18], chicken digesta [19], sediments, bioreactors [20] and cricket hindguts [21], and should therefore be applicable to any environment, especially those where the GC-content of the environmental DNA is not evenly distributed. To our knowledge, this is the first time this technique has been applied in genome-resolved metagenomics, and we demonstrated its suitability to reconstruct medium- and high-quality MAGs from rare (< 0.1% relative abundance) [22] and very rare (< 0.01% relative abundance) [23] microbial taxa in soil, a highly complex environment [24] (see Fig. 1).

## Materials and methods

### Soil samples and DNA extraction

Soil samples were collected from four sites around Scotland (UK). Two sandy loam soil samples, GcA and GcB, with pHs of 4.5 and 7.5, respectively, were collected from a pH-controlled agricultural soil (Scottish Agricultural College, Craibstone, Scotland) that had been maintained for >60 years [25]. A humus-iron podzol soil sample, GcL, was collected from an acidic marsh and a peaty gleyed podzol soil sample, GcC, was collected from an acidic moorland (Table S1). DNA was extracted from 0.5 g of soil as described previously [26]. Three DNA extractions were performed per soil sample, pooled, and cleaned using the DNeasy Power CleanUp Kit according to the manufacturer’s instructions (Qiagen). The resulting DNA quality was assessed by spectrometry (Nanodrop ND-1000) and with 1% agarose gel electrophoresis.

### GC fractionation

DNA was then fractionated based on its GC-content using a modified version of a published protocol [18, 27]. Specifically, bisbenzimide (Hoechst 33258) (1 mg/ml) was added to a CsCl solution (refractive index = 1.3970, containing 5 mM Tris, pH 8.0) in a 1:500 (v/v) ratio. One μg of high-quality DNA was added to an 8 ml ultracentrifuge tube (Quick-Seal® Bell-Top Polypropylene Tube, Beckman) before filling the tube with CsCl solution and mixing thoroughly. The ultracentrifuge tubes were then balanced by pair, sealed, and subjected to centrifugation (45 000 rpm (equivalent to 98 318 average g-force) at 4°C for 56 h) in an Optima MAX-XP ultracentrifuge using an MLN-80 rotor (Beckman Coulter). The resulting gradient in the ultracentrifuge tube was fractionated into 16 fractions of 500 μl each using a fraction recovery system (Beckman Coulter). Each fraction’s refractive index was measured using an AR200 Refractometer, Reichert™. Each fractionated sample was mixed with an equal volume of isopropanol followed by a brief centrifugation (16 000 g for 1 min) before discarding the top aqueous layer. This washing procedure was repeated two times to remove excessive bisbenzimide [18]. Each sample was then mixed with twice the volume of PEG solution (30% PEG 6000 and 1.6 M NaCl) and 1 μl glycogen at 4°C overnight, followed by pelleting the DNA with a 16 000 g centrifugation at 4°C for 45 min. The aqueous phase was then discarded, and the DNA pellet was washed with 1 ml of 70% ice-cold ethanol. After a 16 000 g centrifugation at 4°C for 15 min, the ethanol was discarded, and the DNA pellet was dried at 55°C for 1 min before being eluted in 30 μl of molecular biology grade water. All DNA samples were stored at −80°C before further use.

### Modelling the average GC-content from the refractive index using pure cultures of bacteria

Three bacterial pure cultures with known GC-content were included, namely Streptococcus uberis NCIMB 702055 (GC-content = 36.6%), Escherichia coli K12 (GC-content = 50.6%) and Micrococcus luteus NCIMB 9278 (GC-content = 73%) (Table S2). DNA extraction of 2-ml fully grown cultures was performed as described previously [28] for the three bacterial pure cultures.

**Figure 2:**
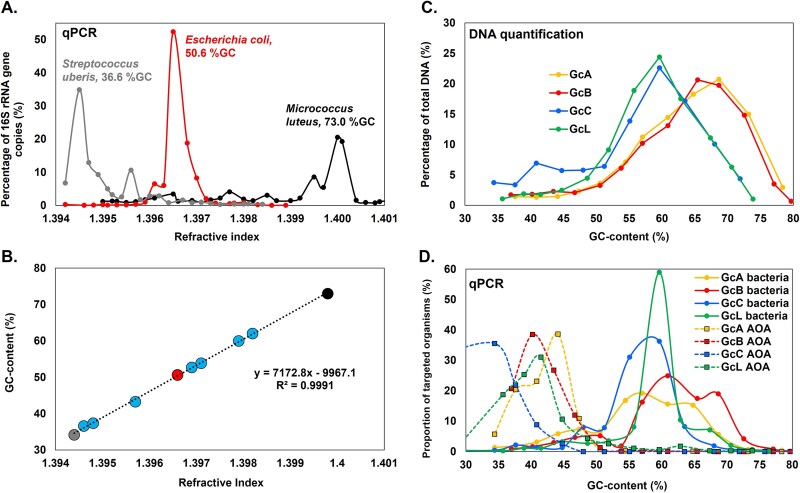
Molecular separation of DNA based on GC-content using a bisbenzimide-CsCl DNA fractionation approach. (A) The distribution of the DNA from eight bacterial and three archaeal species across the fractions was determined by qPCR and their refractive index of the fractions was determined. The distribution of DNA from three bacterial species is shown for reference. (B) The refractive index of fractions possessing the DNA of eight bacteria and three archaea was plotted against the known GC-content of their genomes. The relationship between the observed refractive index and their GC-content was determined as per the presented equation. The three colored dots (grey, red, and black) correspond to the bacteria represented in panel A for reference. (C) Amount of DNA across GC-content fractions of the four soils (GcA, GcB, GcC, and GcL). (D) Relative abundance of the bacterial 16S rRNA and archaeal *amoA* genes across GC-content fractions of four soils (GcA, GcB, GcC, and GcL), estimated based on the 16S rRNA and archaeal *amoA* gene qPCR on each fraction, respectively. For each soil, 100% represents the total number of each marker gene across all GC-content fractions.

The high-quality DNA from the bacterial pure cultures was fractionated using the same protocol described above. The abundance of the 16S rRNA gene was assessed in each fraction by qPCR using bacterial 16S rRNA primers 341F / 907R (see above). A linear model was tested between the DNA GC-content and the refractive index of bisbenzimide CsCl fraction (Fig. 2A and B, Table S2), as described previously [16]. A strong relationship (*R*^2^ = 0.99) existed between the GC-content and the refractive index following equation 1:


(1) \begin{equation*} GCcontent=6489.9\ Refractive\ Index-9013.3 \end{equation*}

### Refining GC-content prediction with a range of bacterial and archaeal pure cultures

The accuracy of the approach was tested using eight bacterial and three archaeal pure cultures, which span a wide range of GC-content (Table S2). In addition to the three previously described bacteria, five other bacteria were included: *Nitrosomonas europea* (GC-content = 50.7%), Bacillus subtilis 168 Δ6 (GC-content = 43.3%), Nitrosospira multiformis (GC-content = 53.9%), Pseudomonas putida F1 (GC-content = 62.0%) and *P. putida* U NCIMB10015 (GC-content = 60.0%). The three selected archaea included *Nitrososphaera viennensis* (GC-content = 52.7%), *Nitrosopumilus maritimus* (GC-content = 34.2%) and *Nitrosotalea devaniterrae* Nd2 (GC-content = 37.4%). Culture media and DNA extraction approaches are described in Table S2. The fractionation procedure used the same protocol as described above.

Another qPCR approach was used to recover both archaea and bacteria, with the universal 16S rRNA primers 515mF (5′- GTG YCA GCM GCC GCG GTA A -3′) [29] and 806mR (5′- GGA CTA CNV GGG TWT CTA AT-3′) [30] due to the enzyme previously used being discontinued by the manufacturer. Here, the 16S rRNA gene qPCR reaction consisted of an initial step at 95°C for 3 min, followed by 40 cycles of 95°C for 15 s, 53°C for 35 s, 72°C for 45 s, and 80°C for 8 s followed by reading, ending with a melting curve analysis. Each 20-μl qPCR reaction consisted of 10 μl iQ SYBR Green™ qPCR master mix (Bio-Rad), 0.2 mg ml^−1^ bovine serum albumin (BSA), 2 μl DNA template, and 0.5 μM of primers. All qPCR assays used the same full-length *E. coli* 16S rRNA gene standard as above.

Another linear model was tested between the DNA GC-content and the refractive index of bisbenzimide CsCl fraction and this model was compared to the three-strain one. Both models were highly correlated (see Fig. S1), so a final linear model (equation 2) was used to estimate the average DNA GC-content in each fraction from the soil DNA, based on its refraction index.


(2) \begin{equation*} GCcontent=7172.8\ Refractive\ Index-9967.1 \end{equation*}

### Microbial quantification by qPCR

For the soil DNA, the bacterial 16S rRNA gene and the archaeal *amoA* gene were quantified in the unfractionated and in each fractionated sample (Table S3). Each 20-μl qPCR reaction consisted of 10 μl QuantiFast™ qPCR master mix (Qiagen), 0.2 mg ml^−1^ bovine serum albumin (BSA), 2 μl DNA template, and the 0.4 to 0.6 μM of primers. The bacterial 16S rRNA gene primers were 341F / 907R [31] and the archaeal *amoA* gene primers were 23F/616R [32]. The 16S rRNA gene qPCR reaction consisted of an initial step at 95°C for 5 min, followed by 38 cycles of 94°C for 15 s, 67°C for 45 s, and 80°C for 8 s followed by reading, ending with a melting curve analysis. The archaeal *amoA* gene qPCR reaction was as described previously [33]. All qPCR assays used a 10-fold serial dilution from 10 to 10^8^ copies of known DNA template as standard. The standard for bacterial 16S rRNA gene qPCR was a full-length *E. coli* 16S rRNA gene PCR product. A cloned archaeal *amoA* gene was used as the standard as reported previously [33]. All qPCR assays had *R*^2^ values 0.98–1, and their efficiencies were between 95% and 101%. While the 16S rRNA primers targeted the 16S rRNA gene of bacteria, the archaeal *amoA* primers were specific to AOA (not encompassing the ammonia-oxidising bacterial *amoA* gene).

### Analysis of 16S rRNA gene amplicon library

For universal 16S rRNA gene amplicon sequencing, triplicate PCR reactions targeting the V4 region were performed per sample. Each 25-μl PCR reaction contained 12.5 μl KAPA HiFi HotStart ReadyMix (Roche), 2 μl formamide, 0.5 μM each of the primers 515mF and 806mR with MiSeq sequencing adaptors, and 1 μl DNA sample as template. The PCR amplification was 95°C for 3 min, followed by 35 cycles of 98°C for 20 s, 50°C for 10 s and 72°C for 15 s, and then a final extension at 72°C for 5 min. The products of triplicate PCR were checked on 1% agarose gel and pooled. The 16S rRNA gene amplicon libraries were performed with the Herculase II Fusion DNA Polymerase Nextera XT Index V2 Kit and were sequenced on MiSeq 300PE by Macrogen company to a depth of 179 356 reads per sample on average.

The 16S rRNA gene amplicon sequences were trimmed with TrimGalore v0.6.6 [34] for trimming of sequencing primers and adapters and FastQC v0.11.9 [35] for quality control. Trimmed reads were merged and assigned to amplicon sequence variants (ASVs) using DADA2 v1.30.0 [36], with quality filtering and chimera removal using the default settings (Table S4). ASV libraries were subsampled to 15 735 reads, which was the read number in the smallest library (Table S4) and singletons were removed. Taxonomy (Table S5) was assigned to ASVs in DADA2 using the Genome Taxonomy Database (GTDB) 16S rRNA database (release R207) -(https://data.gtdb.ecogenomic.org/releases/). Community diversity (Table S6) and taxonomic assignment were calculated using the R package, phyloseq v1.46.0 [37] and these outputs were used to estimate the relative abundance of each family. The relationship between communities was calculated using UniFrac, which compares community composition taking into account phylogenetic relatedness of the ASVs. A Principal Coordinate Analysis (PCoA) plot of the community dissimilarity was generated using the phyloseq package.

### Metagenomic sequencing and genome assembly

DNA libraries were prepared for several selected high and low GC-content fractions (GcA5, GcA13, GcB4, GcB14, GcC5, GcC11, GcL4, and GcL13) using the Nextera XT DNA Library Preparation Kit and sequenced using Illumina NovaSeq 6000 S4 platform (276 million reads per fraction on average, Table S7) by Macrogen company.

Reads were filtered using the READ_QC module [38], and high-quality reads for each metagenome were assembled using MEGAHIT v1.1.3 [39]. Binning of resulting contigs was performed with MaxBin2 [40] and metaBAT2 [41], and the results were consolidated using the Bin_refinement module [38]. Completeness and contamination of bins were estimated with CheckM v1.1.2 [42], and only bins with a completeness of >45% and contamination of <10% were retained for further analysis (Table S8). Bins were initially characterized using the classify_wf function in GTDB-Tk v2.1.1 [43] using the R207v2 GTDB release. The taxonomy described throughout is based on the GTDB taxonomy and may differ from other sources, such as NCBI taxonomy. Genome coverage and MAG relative abundance were calculated using read recruitment with CoverM v0.6.1 [44] in the fractions from which they were assembled. The relative abundances of each genome’s taxonomic family were also estimated in the unfractionated samples and in each fraction using the 16S rRNA community analysis described above.

### Genome characterisation and metabolic prediction

The species tree of the new genomes was created based on the taxonomy provided by GTDB [45]. Novelty of each branch of the tree (defined as the percentage of the total number of genome sequences available for a given taxa that were sequenced in this work) was calculated using the R207v2 GTDB release as a proxy for the diversity of publicly available genomes. Genomes were annotated using Prokka v1.14 [46] and the KEGG database [47] using GhostKOALA [48]. Carbohydrate active enzymes were annotated using profile HMM database dbCAN-HMMdb-V11 [49], filtered with hmmscan-parser.sh and by removing matches with mean posterior probability <0.7. Biosynthetic gene clusters (BGCs) were annotated with antiSMASH v7.1.0 using default settings.

## Results

### Differential microbial distribution across a GC-content gradient

DNA of eight bacteria and three archaea of known genomic GC-content was fractionated using the GC-fractionation procedure (Fig. 2A) and a linear model (Fig. 2B) demonstrated the strong relationship between the GC-content and the refractive index of the fractions. Based on this relationship, the GC-content was estimated for each fraction of the environmental DNA extracted from four UK soil sites (Craibstone pH 4.5 (GcA) and pH 7.5 (GcB), Corsemaul (GcC), and Lower Dell (GcL)) (Table S1) following the GC-fractionation procedure. The DNA was unevenly distributed across the GC gradient for the four soil samples, with fractions with the highest DNA amounts corresponding to GC-contents of 58%–65% for the four soils (Fig. 2C, Table S3). Most of the DNA was present within 5% GC-content from the peak, and less than 12% of total DNA was present in any fraction with a GC-content <55%.

The abundance of the bacterial 16S rRNA and ammonia monooxygenase subunit A (*amoA*) genes were determined in each fractionated DNA sample by target-specific qPCR assays (Table S3). While ubiquitous in many environments, AOA were chosen as rare microbes having low GC-content [7]. Within each unfractionated soil DNA, bacteria were between two and four orders of magnitude more abundant than AOA (Table S3). Bacteria and AOA were differently distributed across the GC-gradient in the four soils, with most bacteria (66%–88%) being present in the GC-content range of 50%–70%, while most of the AOA (93%–100%) were present in the low GC-content range (GC < 50%) (Fig. 2D, Table S3). Therefore, AOA were relatively enriched in the low GC-content fractions compared to the high GC-content fractions where bacteria dominated. Bacterial DNA was concentrated within a single range for the soils GcC and GcL, forming a single peak, but appears to form two peaks on the GC-content gradient for the agricultural soils GcA and GcB (Fig. 2D).

**Figure 3:**
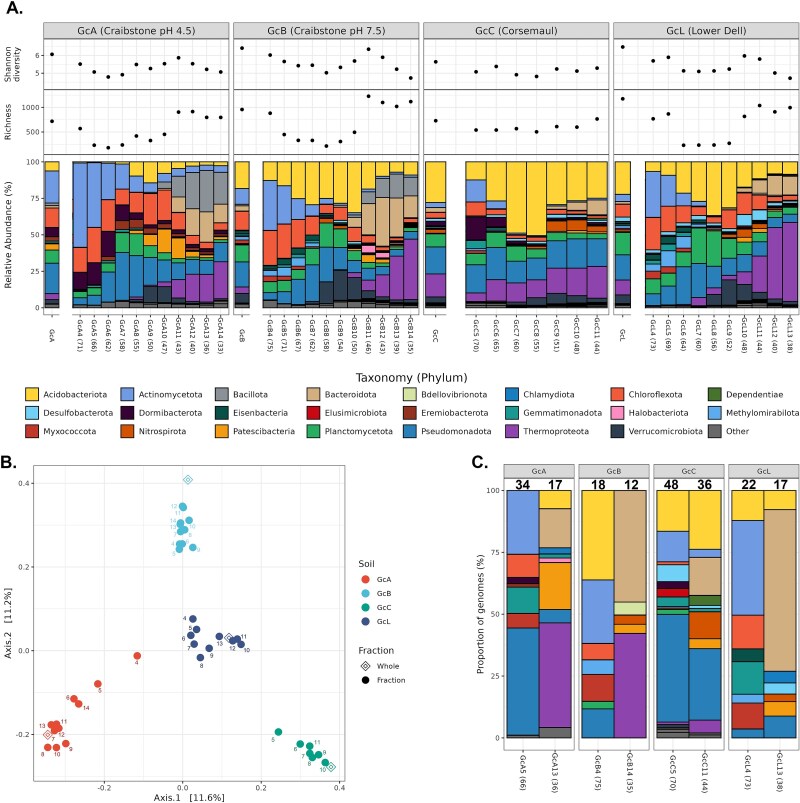
Microbial communities across a GC-content gradient following a bisbenzimide-CsCl GC-content-based DNA fractionation approach in four soils (GcA, GcB, GcC, and GcL). (A) Taxonomic composition, richness and diversity of microbial community in each unfractionated soil DNA and associated fractionated-based DNA. Richness is defined as the total number of ASVs detected. GC-content percentage of each fraction is indicated in brackets following the fraction name. (B) Principal coordinate analysis of unweighted Unifrac using the microbial composition (ASV-based) of each fraction. The number next to each dot indicates the fraction number of each sample. (C) Taxonomy of metagenome-assembled genomes recovered from each fraction. Numbers above the bars indicate the number of genomes recovered from each fraction. Numbers below the bars indicate the fraction name, followed by the GC-content percentage of the fraction in brackets.

Universal 16S rRNA gene sequencing was performed (MiSeq V3 sequencing, Illumina) on each fractioned and unfractionated DNA sample of the four soils (Table S4). Even at the phylum level, there was an observable difference in the microbial composition of each soil along the GC-content gradient. Fractions from both extremes of the gradient possess different communities from the original soils (Fig. 3A, Table S5), and archaea represent at higher relative abundance in the low-GC fractions of three soils (Fig. S2). These results also indicate that the two peaks observed for GcA and GcB in Fig. 2D are the result of the fractionation procedure separating bacterial phyla that are dominant in the unfractionated samples, notably Pseudomonadota and Actinomycetota. There was a trend for higher diversity in the lower GC-content fractions (Fig. 3A, Table S6), although most of the DNA was present in the 50%–70% GC-content range (Fig. 2C). Therefore, low GC fractions are likely an enriched source of DNA for rare microbes. A PCoA plot of the community dissimilarity also indicates compositional change between unfractionated and fractionated samples (Fig. 3B). GC-fractionated pseudo-communities from each site are dissimilar to each other, but are more similar to their original communities rather than to pseudo-communities of other sites with similar GC-content (Fig. 3B).

### Novelty of genome-resolved metagenomes of rare microbes

Metagenomic sequencing (NovaSeq (Illumina)) was performed on one high and one low GC-content fraction per soil (Table S7). A total of 204 MAGs (completeness >45% and contamination <10%) could be reconstructed from the eight metagenomes: 122 genomes from high GC-content fractions and 82 genomes from low GC-content fractions (Table S8). The MAGs possessed an average completeness of 67% (range 45%–99%) and an average contamination of 4% (range 0%–10%). On average, each genome was present at 0.34% relative abundance in its fractionated pseudo-community (range 0.07%–2.67%) (Table S8). Notably, 31% of genomes were from rare or very rare microbial families (Table S9). The GC-content of most of these genomes corresponded with the GC-content fractions, with high and low GC-content genomes recovered from high and low GC-content fractions, respectively (Fig. S3, Table S10), indicating that GC-content based separation of DNA was successfully achieved for these soils. There was no significant difference in the quality of genomes (based on criteria of completeness and contamination) recovered from the high and low GC-content fractions (Fig. S4).

**Figure 4:**
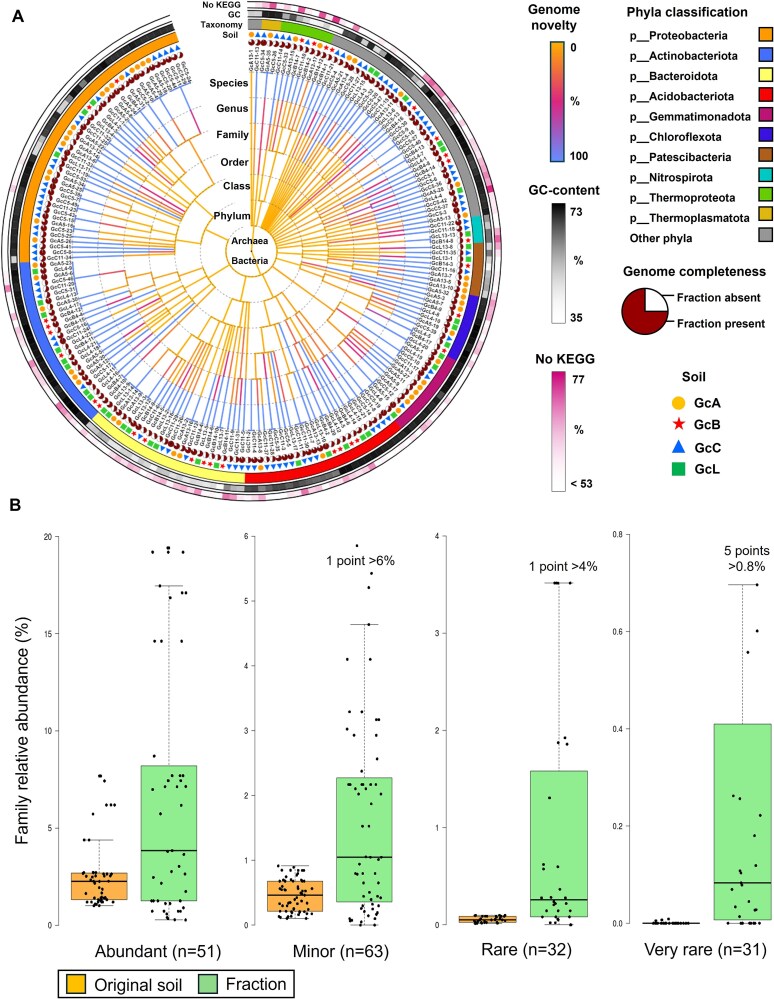
Recovered metagenome-assembled genomes (MAGs) from the low- and high-GC-content fractions in the four soils. (A) Taxonomy, novelty and genomic characteristics of 204 recovered metagenome-assembled genomes. The curated genome database GTDB R207v2 is used as a proxy to represent publicly available genomes. Phyla with less than four genomes recovered in this study are classified as “other phyla” in this figure. “No KEGG” indicates the percentage of genes with no close homologs in the KEGG database. Details of each MAG and genome novelty of each branch are available in Tables S8 and S11, respectively. (B) Relative abundance of genome families in the original and fractionated soil DNAs for families being abundant (> 1%), minor (< 1%), rare (< 0.1%), and very rare (< 0.01%) in the original soil DNA. The family taxonomy is assessed based on the genome sequence, while the relative abundances are determined through 16S rRNA gene amplicon sequencing analysis. The number of genome families (n) in each category is indicated. P-values indicate statistical differences between the two groups (t-test 1-tail paired) for each category. A low amount of data within the fractionated samples was omitted from the graphical representation (i.e. one, one, and five values for the minor, rare, and very rare categories, respectively) because they exceeded the maximum value of the y-axis. Detailed family abundance information for each recovered genome is available in Table S9.

The 204 MAGs were taxonomically diverse, representing 27 bacterial and four archaeal phyla (Table S11, Fig. 3C) based on the GTDB classification (release R207v2) [50]. Seventy genomes were from previously uncharacterized genera (Fig. 4A; Table S8), whereas the reconstructed MAGs also increased the representation of 24 known genera by over 50%, and almost all MAGs were from novel species (202 of 204 genomes) (Table S8). Most MAGs (61%) recovered in this work are from families that were enriched in the GC-based fractions compared to original soils (enrichment range: 1.1–534.4 -fold) (Table S12), and this molecular enrichment was statistically significant in rare (*P* = 3.3 × 10^−4^) and very rare (*P* = 2.7 × 10^−3^) microbial families (Fig. 4B).

The MAGs sequenced in this study possessed a high percentage of genes with no close homologs in the KEGG database. The average percentage of uncharacterized genes per genome was 55% (range 30%–78%) (Table S8). Despite this, genes involved in important biogeochemical cycles and carbohydrate degradation were often detected (Tables S13 and S14). In total, 670 biosynthetic gene clusters (BGCs) were detected in the 204 genomes, with most genomes (76%) possessing at least one BGC. Ribosomally synthesized and post-translationally modified peptides were the most frequently detected category of BGC (32%), followed by polyketide synthases (20%), terpene (19%), non-ribosomal peptides (18%), and other BGCs (10%) (Table S15). There was no significant difference between the number of BGCs in genomes from different abundance ranks. Finally, BGCs were mostly not retrieved in the genomes of certain taxa, such as the Chloroflexota family CSP1–4 and the Patescibacteria phylum (Table S15).

### Rare biosphere genomes of the Bacteroidia

As the majority of DNA was present in the higher GC-content fractions, the lower GC-fractions were more likely to contain DNA from rarer microbial families. Notably, 19 genomes sequences were recovered from the low-GC content possessing Bacteroidia class from the orders Chitinophagales (10 genomes), Sphingobacteriales (3 genomes), and Flavobacteriales (1 genome), and the uncharacterized orders o__AKYH767 (3 genomes), o__AKYH767-A (1 genome), and o__NS11–12 g (1 genome). Seven of these genomes were from rare families (< 0.1% relative abundance), and six were from very rare families (< 0.01% relative abundance) that were enriched 3.2 to 56.8 -fold, compared to soils (Table 1).

**Table 1:** Recovered Bacteroidia genomes, family enrichment score following GC-fractionation procedure and family abundance rank in the original, unfractionated sample. The genome completeness, genome contamination scores as well as taxonomy (order, family, and genus) are indicated for each MAG. In addition, the relative abundance of each genome’s family in the unfractionated sample and in the fraction of interest are indicated. For the family abundance rank, each family was classified as abundant (> 1%), minor (0.1%–1%), rare (0.01%–0.1%), or very rare (< 0.01%), based on its 16S rRNA gene relative abundance in the unfractionated sample. For each genome’s family, the amount of enrichment resulting from the GC fractionation is represented by the ratio of the 16S rRNA gene relative abundance in the fraction of interest compared to the unfractionated sample.

MAG name	Completeness (%)	Contamination (%)	Order	Family	Genus	Relative abundance in unfractionated DNA (%)	Abundance rank	Relative abundance in fraction of interest (%)	Fold family enrichment
GcB14–4	87	3	o__AKYH767-A	f__OLB10	g__	0.09	Rare	0.28	3
GcL13–5	66	0	o__NS11–12 g	f__UKL13–3	g__CAIUEQ01	0.00	Very rare	0.08	*
GcB14–6	52	3	o__Flavobacteriales	f__UBA2798	g__	0.00	Very rare	0.03	*
GcA13–2	81	2	o__Sphingobacteriales	f__Sphingobacteriaceae	g__Mucilaginibacter	0.55	Minor	3.17	6
GcA13–16	62	5	o__Sphingobacteriales	f__Sphingobacteriaceae	g__Mucilaginibacter	0.55	Minor	3.17	6
GcC11–12	83	3	o__Sphingobacteriales	f__Sphingobacteriaceae	g__Mucilaginibacter	0.02	Rare	1.31	57
GcC11–29	57	2	o__AKYH767	f__B-17BO	g__PALSA-968	0.10	Rare	4.51	48
GcC11–26	99	0	o__AKYH767	f__Palsa-965	g__Palsa-965	0.00	Very rare	0.26	*
GcL13–16	48	1	o__AKYH767	f__Palsa-948	g__Palsa-948	0.01	Very rare	0.26	51
GcB14–5	54	0	o__Chitinophagales	f__BACL12	g__UBA7236	0.00	Very rare	0.07	*
GcL13–2	48	2	o__Chitinophagales	f__UBA10324	g__JAFDYS01	0.00	Very rare	0.01	*
GcB14–9	89	4	o__Chitinophagales	f__Chitinophagaceae	g__VBAT01	0.91	Minor	5.21	6
GcC11–6	51	0	o__Chitinophagales	f__Chitinophagaceae	g__Flavipsychrobacter	0.39	Minor	0.51	1
GcL13–3	46	3	o__Chitinophagales	f__Chitinophagaceae	g__UTBCD1	0.09	Rare	3.52	40
GcL13–12	87	0	o__Chitinophagales	f__Chitinophagaceae	g__UTBCD1	0.09	Rare	3.52	40
GcL13–14	55	7	o__Chitinophagales	f__Chitinophagaceae	g__Puia	0.09	Rare	3.52	40
GcA13–14	81	4	o__Chitinophagales	f__Chitinophagaceae	g__Puia	1.01	Abundant	17.10	17
GcA13–9	59	8	o__Chitinophagales	f__Chitinophagaceae	g__Puia	1.01	Abundant	17.10	17
GcL13–9	70	6	o__Chitinophagales	f__Chitinophagaceae	g__Puia	0.09	Rare	3.52	40

^*^Family was detected in fraction, but was too low abundance to be detected in original soil.

As noted for the Bacteroidia families Bacteroidaceae and Flavobacteriaceae, these microbes appear to not form endospores [51, 52], as they lack genes for endospore formation (Table S16). All Bacteroidia recovered in this study are predicted to be non-motile as they lacked genes for chemotaxis and flagellar assembly (Table S16). Most Bacteroidia genomes possess marker genes for aerobic respiration (*coxA*, *coxB*, *ctaA*, and *ctaB*), and several genomes also have genes for microaerobic respiration terminal oxidases, cytochrome bd ubiquinol oxidase (*cydA* and *cydB*) [53–55], and cytochrome cbb3 oxidase (*ccoN*, *ccoNO*, *ccoO*, and *ccoP*) [56] (Table S16). Additionally, they lack evident marker genes of a strictly anaerobic lifestyle, such as fumarate hydratase class 1 (*fumA* and *fumB*) [57] and pyruvate formate lyase (*pflD*) (Table S16). Together this indicates that these organisms are adapted to aerobic or microaerobic lifestyles.

Lactate is a key metabolite in microbial cross-feeding in the human gut [58–61] and its utilization significantly influences colonic ecosystem pH, microbial metabolism, and community stability [60]. In contrast, the role of lactate cycling in soils is less well understood, and its contribution to soil microbial ecosystems remains an open question. Most of the Bacteroidia genomes possess a three-component- type L-lactate dehydrogenase [62], critical for converting L-lactate to pyruvate. Only one genome, GcL13–9, possesses a lactate permease, which has been predicted to be necessary for utilising exogenous lactate [63]. Manual curation of the genomic region surrounding the three-component L-lactate dehydrogenase genes in GcA13–16 revealed the presence of an L-fucose permease, and an L-fucose degradation pathway that produces L-lactate as an end product (Table S17). The L-lactate dehydrogenase was present in many of the reconstructed Bacteroidia genomes (Table S17), indicating that this enzyme may, in fact, function in utilising intracellularly produced lactate as part of L-fucose utilisation.

## Discussion

The rare biosphere makes up a significant portion of the diversity of our planet [64]. Many studies focus on the more abundant members of microbial communities, but low-abundance microbes also drive some critical ecosystem functions, especially when performing metabolic activities that are taxonomically restricted, such as in the case of microbial ammonia oxidation [65]. While it is difficult to reconstruct genomes from rare environmental microbes with the classical metagenomic approach, we demonstrated the potential of GC-fractionation to overcome this gap. In this proof-of-concept work, 63 genomes were recovered from rare or very rare microbial families. It is unclear whether these low-abundance organisms are naturally ecologically constrained to a low abundance by virtue of their biochemical processes or whether they act as a “functional seed bank” [10], being only transiently in low abundance and increasing in abundance under certain environmental stimuli, such as acidic/osmotic stress or the intermittent introduction of a specific nutrient into the ecosystem. Recent co-occurrence network analysis suggests that low-abundance taxa have stronger ecological relevance to the community than more abundant taxa in terrestrial ecosystems [66], but a mechanistic explanation for this is lacking. Obtaining genome sequences for these rare taxa would enable a greater knowledge of their metabolic potential and role in soil microbial ecosystems. Those low-abundance taxa may represent habitat specialist groups, in contrast to the more abundant microbes being habitat generalists [66–68], even if a wide range of natural abundance has been observed for generalists [69, 70].

More than half of the reconstructed genomes (124 out of 204) belonged to families whose 16S rRNA gene relative abundance was increased from the unfractionated to the fractionated samples. While family enrichments up to 534-fold could be calculated, it is also notable that 20 genomes were assembled from families that were not detected in the corresponding unfractionated soil, preventing calculation of the enrichment of these families. However, based on the number of amplicons sequenced in the unfractionated samples (subsampled to 15 735) and the abundance of these families in the fractionated samples, it appears that many of these genomes belong to families that have been enriched to an even greater degree. Notably, the Dormibacteria family f__UBA8260 was potentially enriched more than 839-fold in the Corsemaul (GcC5) high-GC fraction, compared to the unfractionationed sample (GcC). Taking these genomes into account in the overall family enrichment score, ~60% of the reconstructed genomes belong to families that have been selectively enriched using the described GC-fractionation approach.

The recovered genomes presented extensive taxonomic diversity, especially for the low GC-content MAGs, representing a diverse pool of rare microbes. Among the recovered genomes, several genomes represented novel orders (e.g. GcC11–19, novel order within the Gammaproteobacteria class, and GcA5–32, novel order within the UBA5177 class), novel families (14 novel families), and numerous novel genera (67 genomes belonging to novel genera) (Table S8). Reconstruction of previously undiscovered genomic diversity is critical to advancing evolutionary questions, such as those related to microbial diversification rates [71, 72] or the origins of eukaryotes [73, 74]. In addition, the reconstructed genomes included 19 genomes belonging to the Bacteroidia class, allowing some metabolic inference of this group. Such genome reconstruction could also further clarify the ecological strategy of microbes, as low GC genomes were reported to follow oligotrophic strategies, while high GC genomes are copiotrophs [75]. This demonstrates that this selective sequencing approach enables reconstruction of multiple genomes from specific taxonomic groups. Therefore, such increased genomic representation allows metabolic inference of poorly studied microbial groups and deeper understanding of the mechanisms of microbial evolutionary genomic dynamics [7, 76–78].

## Supplementary Material

20250731_GC_fract_SI_text_ycaf152

20250610_GC_fract_SI_tables_ycaf152

## Data Availability

Scripts for general manipulation of the 16S rRNA gene diversity have been deposited at https://github.com/bodington/gc_16S/, and scripts specific to the genome novelty have been deposited at https://github.com/SheridanPO-Lab/Genome-novelty. The 16S rRNA gene sequencing data and the 204 new genome sequences presented in this work have been deposited under the NCBI BioProject PRJNA1160233.
