# Supplementary material for: Genomic recovery from rare terrestrial microbes enabled by DNA-based GC-fractionation
Source: ISME Commun. 2025 Sep 3;5(1):ycaf152. doi: 10.1093/ismeco/ycaf152 (PMC12477608; doi:10.1093/ismeco/ycaf152)
Supplement: 20250731_GC_fract_SI_text_ycaf152 [file 20250731_gc_fract_si_text_ycaf152.pdf]

**Supplementary Information: Genomic recovery from rare terrestrial microbes enabled by DNA-based GC-fractionation**

Paul O. Sheridan[1,2], Yiyu Meng[1], Dylan Bodington[1], David Coutts[1],Tom A. Williams[3], Cécile Gubry-Rangin[1,#]

**Author affiliations:**

[1]School of Biological Sciences, University of Aberdeen, UK.

[2]School of Biological and Chemical Sciences, University of Galway, Ireland.

[3]School of Biological Sciences, University of Bristol, UK.

[#]Corresponding author: Cécile Gubry-Rangin. c.rangin@abdn.ac.uk

**Supplementary Figures:**

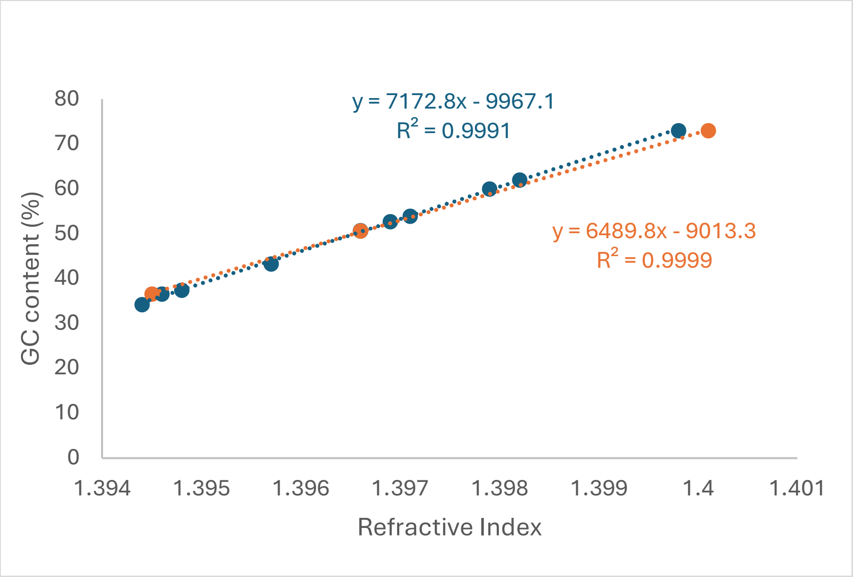


**Figure S1: The relationship between the observed refractive index and its GC-content was determined as per the two presented equations. The model in orange corresponds to three bacteria, while the model in blue represents eight bacteria and three archaea.**

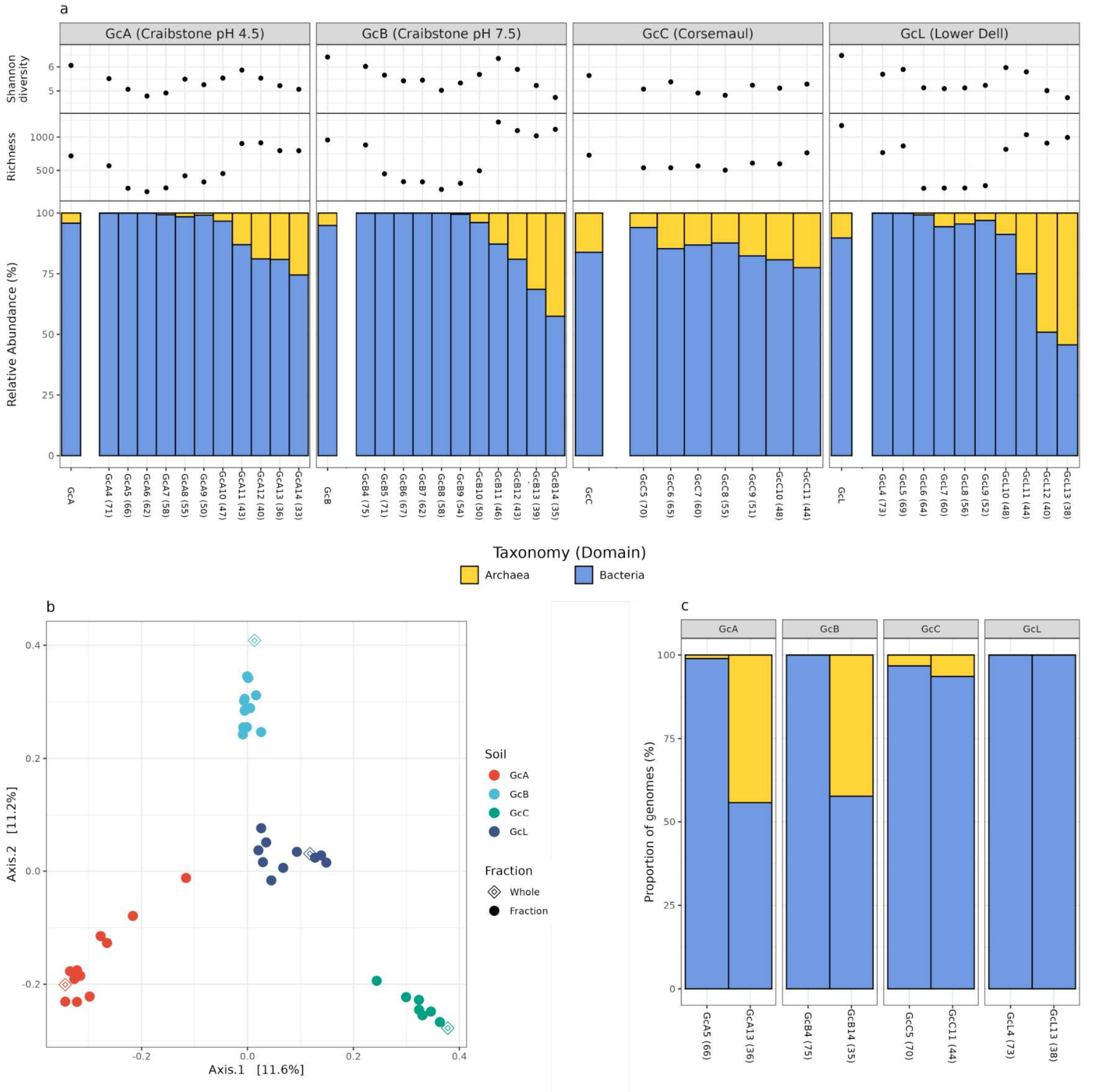


**Figure S2: Domain-level microbial communities across a GC-content gradient following a bisbenzimide-CsCl GC-content-based DNA fractionation approach in the four soils (GcA, GcB, GcC and GcL). A** Taxonomic composition, richness and diversity of each soil DNA and associated fractionated-based DNA. Richness is defined as the total number of ASVs detected. GC-content percentage of each fraction is indicated in parenthesis following the fraction name. **B.** Principal coordinate analysis of unweighted Unifrac using the microbial composition of each fraction. **C.** Taxonomy of metagenome-assembled genomes recovered from each fraction.

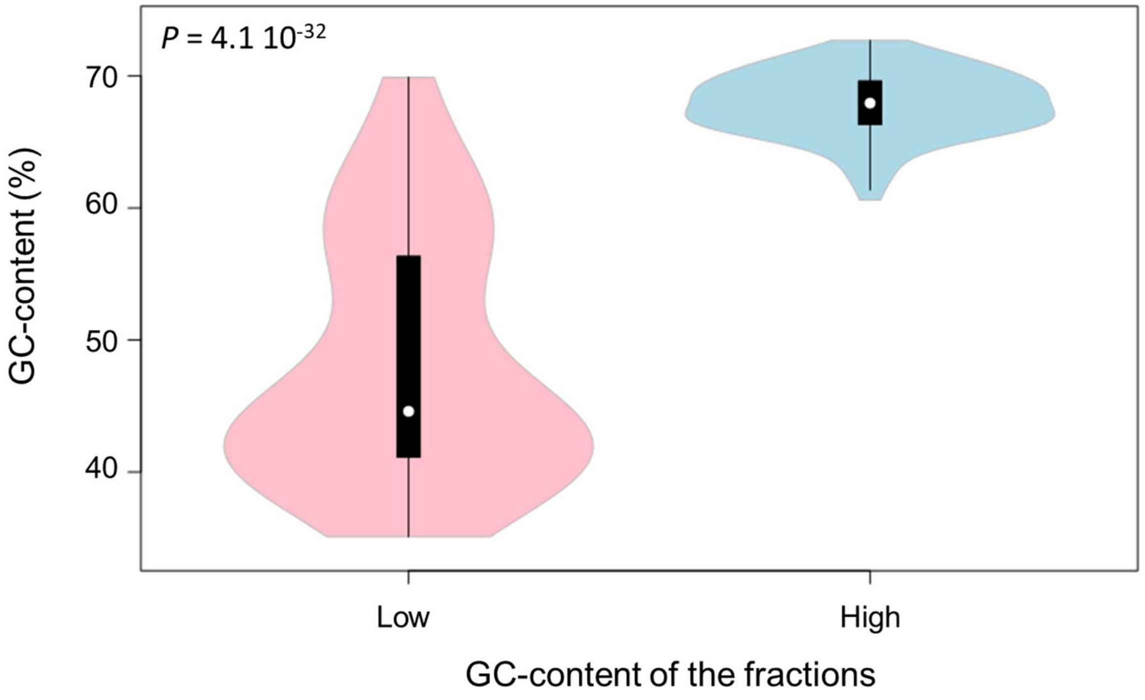


**Figure S3: GC-content of low and high fraction genomes.** *P* value indicates statistical difference between the two groups (t-test 1-tail unequal variance).

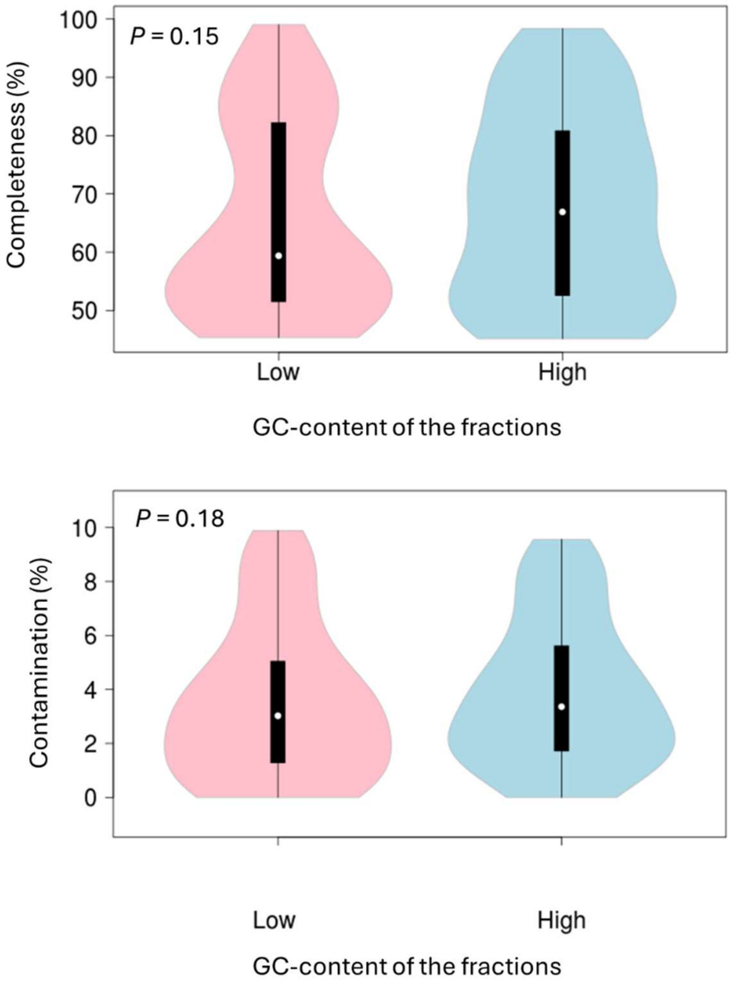


**Figure S4: Genome completeness and contamination of low and high fraction genomes.** *P* value indicates no statistical difference between the low and high fraction genomes for either genome completeness or contamination (t-test 1-tail unequal variance).
